# A high-throughput quantification of resin and rubber contents in *Parthenium argentatum* using near-infrared (NIR) spectroscopy

**DOI:** 10.1186/s13007-019-0544-3

**Published:** 2019-12-17

**Authors:** Zinan Luo, Kelly R. Thorp, Hussein Abdel-Haleem

**Affiliations:** US Arid-Land Agricultural Research Center, USDA-ARS, Maricopa, AZ 85138 USA

**Keywords:** *Parthenium argentatum*, Guayule, Resin, Rubber, Near-infrared (NIR) spectroscopy, Partial least squares regression (PLSR), Bioenergy crop

## Abstract

**Background:**

Guayule (*Parthenium argentatum* A. Gray), a plant native to semi-arid regions of northern Mexico and southern Texas in the United States, is an alternative source for natural rubber (NR). Rapid screening tools are needed to replace the current labor-intensive and cost-inefficient method for quantifying rubber and resin contents. Near-infrared (NIR) spectroscopy is a promising technique that simplifies and speeds up the quantification procedure without losing precision. In this study, two spectral instruments were used to rapidly quantify resin and rubber contents in 315 ground samples harvested from a guayule germplasm collection grown under different irrigation conditions at Maricopa, AZ. The effects of eight different pretreatment approaches on improving prediction models using partial least squares regression (PLSR) were investigated and compared. Important characteristic wavelengths that contribute to prominent absorbance peaks were identified.

**Results:**

Using two different NIR devices, ASD FieldSpec^®^3 performed better than Polychromix Phazir™ in improving R^2^ and residual predicative deviation (RPD) values of PLSR models. Compared to the models based on full-range spectra (750–2500 nm), using a subset of wavelengths (1100–2400 nm) with high sensitivity to guayule rubber and resin contents could lead to better prediction accuracy. The prediction power of the models for quantifying resin content was better than rubber content.

**Conclusions:**

In summary, the calibrated PLSR models for resin and rubber contents were successfully developed for a diverse guayule germplasm collection and were applied to roughly screen samples in a low-cost and efficient way. This improved efficiency could enable breeders to rapidly screen large guayule populations to identify cultivars that are high in rubber and resin contents.

## Background

Guayule (*Parthenium argentatum* A. Gray), commonly grown in semi-arid regions, is a promising crop to produce natural rubber (NR). NR cannot be replaced completely by synthetic rubber because NR possesses high-performance properties in resilience, impact resistance, abrasion, and heat dispersion, among other desirable properties [1–3]. Almost all the current NR in the US is imported from countries in southeastern Asia, where *Hevea brasiliensis* is widely planted. To increase NR production to meet increasing demands, stabilize economics, and avoid disease threats to *Hevea* in Southeast Asian countries, guayule is considered to be a top alternative resource for domestic rubber production. Additionally, guayule can generate NR latex with much lower Type I *Hev*-b protein, which is important to reduce allergic reactions to medical products–a major problem in the application of *Hevea* rubber [2, 4].

Resin and rubber are the two major industrial components in guayule, which are obtained using a sequential solvent extraction protocol in two steps: treating with polar solvent (acetone, ethanol) to extract resin followed by a non-polar solvent (hexane, cyclohexane, chloroform, etc.) to extract rubber [2, 5–8]. Accelerated solvent extraction (ASE) has been used in analytical chemistry in recent years to accurately determine chemical components [9]. The application of ASE to quantify resin and rubber content in guayule has been previously published [2, 10]. Compared to other solvent-based methods such as Soxhlet and a high-speed homogenizer (Polytron) [5, 6], ASE shortened extraction time by using high temperature and nitrogen pressure while requiring low solvent volumes [2, 8, 11]. However, despite these improvements, using traditional wet chemistry methods to determine chemical contents are time-consuming, labor-intensive, and expensive [12]. The methods to quantify the chemical compositions after extraction procedures are not easily scaled-up to hundreds or thousands of samples, which is the level required for germplasm evaluation in plant breeding programs. Thus, inexpensive, high-throughput, and rapid quantification methods are needed to determine biopolymer components for guayule genetic improvement.

Near-infrared (NIR) spectroscopy, based on vibration properties of organic molecule chemical bonds and their interactions with NIR radiation, is a technique used for rapid, reliable, and non-destructive prediction of chemical components in plants, animal products, food, and pharmaceuticals [12–16]. In the last several decades, NIR spectroscopy has been applied to determine resin and rubber content in guayule [2, 4, 6, 17, 18]; however, these studies were either too early to use advanced multivariate data analysis approaches or the sample size of varieties/accessions was small with a very limited range of rubber and resin contents. Moreover, no previous studies made comparisons between different NIR instruments with varying spectral ranges and resolution. Given these limitations, the objectives of this study were to (1) develop PLSR models using NIR spectroscopy to estimate rubber and resin content for a guayule germplasm containing 56 different accessions; (2) identify optimal pretreatment approaches and ranges of wavelengths for obtaining the most robust and reliable PLSR models; and (3) compare two NIR spectral instruments in prediction accuracy of PLSR models for the estimation of rubber and resin content.

## Materials and methods

### Plant Materials

A total of 49 and 56 guayule accessions (49 were included in 56 accessions) from a USDA germplasm collection were planted under water-stressed and non-stressed field conditions, respectively for 2.5 years with each accession replicated three times [19]. Finally, a total of 315 guayule samples were harvested from water-stressed (147) and non-stressed (168) field plots at Maricopa, Arizona, USA. Trials were irrigated differentially to reach suitable stress levels following the soil water depletion model described by Hunsaker and Elshikha [20]. Two homogenous plants from each plot were harvested in spring of 2018. Harvested plants were dried in an open area then chipped using Troy-Bilt Model 47321 Chipper/Shredder (Garden Way, Inc., Troy, New York) with a 9.53-mm round-holed screen. After drying, the chipped samples were ground using a hammer mill with a 6-mm screen (Model W6H, Schutte-Buffalo Hammermill, LLC, Buffalo, NY). The samples were then fine-ground using a Model 4 Wiley mill to pass the material through 2-mm sieves (Thomas Scientific, Swdesboro, NJ). The dried and ground samples were stored in small sealed plastic bags at 4 °C to limit risk of oxidation.

### Accelerated solvent extraction (ASE) for rubber and resin quantification

Fine dried ground samples weighing 1 ± 0.0005 g were loaded into stainless steel cells (11 mL) of an ASE (Model 200, Dionex Corp., now ThermoFisher Scientific Inc., Waltham, MA), which was equipped with an auto-sampler carousel, a solvent controller that accommodated up to four different solvents, and a collection tray that allowed up to 24 samples to be sequentially extracted [2, 10]. The entire machine was connected to a nitrogen tank. All ASE extraction cells were prepared uniformly. A cellulose microfilter (20-mm diameter) was first placed at the bottom of each cell, which was then filled with dry ground samples mixed with diatomaceous earth (DE). Glass collecting vials (250 mL) were placed into the collection tray. The first cell, as a control, was only filled with DE. Extraction was performed under the following conditions (Table 1): Each sample was first extracted with acetone at 100 °C and 1500 psi of nitrogen, with a heating time of 5 min, static extraction time of 10 min, purge time of 60 s, and flush volume 100%, followed by cyclohexane extraction at 140 °C under the pressure of 1500 psi of nitrogen, heating time of 7 min, static extraction for 20 min, purge time of 60 s, and a flush volume of 100%. Three static cycles were applied to each extraction stage. Following this, the extractant was transferred into a pre-weighed glass vial (250 mL). Evaporation of the solvent from the extract was done in a fume hood at room temperature for 2 weeks and dried in an oven at 55 °C for 24 h before weighing again. Three samples were randomly selected from each ASE batch (11 samples) for moisture content estimation, which was determined by drying a 5-g sample at 105 °C in an oven for 24 h, and then kept 8 h in a desiccator before weighing. The moisture content values of each batch were averaged and used to adjust rubber and resin contents for further use with the following adjustment formula:

1 $${\text{Adjusted resin}} = {\text{\% dry resin content }} \times \left( {1 - {\text{\% moisture content}}} \right)$$

2 $${\text{Adjusted rubber}} = {\text{\% dry rubber content }} \times \left( {1 - {\text{\% moisture content}}} \right)$$

**Table 1:** Two-step accelerated solvent extraction (ASE) method for the extraction of resin and rubber in *Parthenium argentatum*

Preheat	0 min	Pressure	1500 psi
Step 1: acetone extraction			
Heat	5 min	Temp	100 °C
Static	10 min	Acetone	100%
Flush%	100%	Cyclohexane	0
Purge	60 s	Cycles	3
Step 2: cyclohexane extraction			
Heat	7 min	Temp	140 °C
Static	20 min	Acetone	0
Flush%	100%	Cyclohexane	100%
Purge	60 s	Cycles	3

### NIR spectroscopy analysis

Five near-infrared (NIR) spectral scans were collected for each dry ground sample using an ASD FieldSpec^®^3 spectrophotometer (Malvern Panalytical, Cambridge, UK) and a handheld Polychromix Phazir™ model Phazir 1624 spectrophotometer (Polychromix Inc., Wilmington, MA, USA) under ambient temperature. The dry ground samples were stirred and remixed during the scanning intervals. For the ASD scans, the “Muglight” attachment was used with the spectrophotometer, which provided a light source and specialized tray for holding samples during spectral data collection. For the Polychromix device, samples were placed in a plastic laboratory boat with the instrument resting on top of the sample. Standard reference targets were scanned after scanning every fifth sample and tenth sample for ASD FieldSpec^®^3 and Polychromix Phazir™, respectively. The reference target for the ASD was a small 99% Spectralon disk designed to fit in the sampling tray of the Muglight attachment. For the Polychromix Phazir™, the reference target covered the bottom of a weighing pan to avoid light leaking as provided by the manufacturer and used according to the manufacturer’s recommendation. Spectral data of 2151 wavelengths were obtained from the ASD FieldSpec^®^3 with the reflectance ranging from 350 to 2500 nm at 1 nm interval, while spectral data of only 100 wavelengths were obtained from the Polychromix Phair™ with reflectance ranging from 1600 to 2400 nm at 8 nm intervals. As for the reflectance spectra obtained from the ASD FieldSpec^®^3, only the wavelengths between 750 and 2500 nm were used for further analysis since this range covers the NIR region.

### Chemometrics and data analysis

#### Spectral data pretreatment

The Unscrambler X^®^ software (v.10.5, Camo Software AS) was used to perform data pretreatment and establish partial least squares regression (PLSR) models for rubber and resin contents. As a first step to identify and remove outliers, the spectral data was subjected to principal component analysis (PCA). PCA provided a score plot to show the degree of similarity and difference among the samples [21]. From PCA, Hotelling’s T^2^ and Q-residuals explained how far a projection of the sample is away from the origin, and whether the pattern of variables for a sample deviates largely from the model [22]. The samples with both high Hotelling’s T^2^ values and Q-residuals (if any) were detected as outliers and removed before further analysis. Spectral pretreatments were intended to suppress various adverse effects coming from physical properties of the sample, technical errors during measurements or, simply, instrument noise [21]. In our experiment, eight different types of pretreatments were applied to the spectral data to test and compare their effects on the performance of PLSR models, particularly through improvements in the signal-to-noise ratio and in the prediction accuracy. These eight different pretreatments included the following: smoothing using a median filter with segment size of 3, normalization by the mean, baseline correction, standard normal variate (SNV), de-trending (DT) with polynomial order of two, and Savitzky-Golay (SG) first and second derivative calculation. The SG 1st and 2nd derivatives with the window size of 11 (smoothing points = 23) were applied for the spectra obtained from ASD FieldSpec^®^3, and the SG 1st and 2nd derivatives with a window size of 6 (smoothing points = 13) were applied for Polychromix Phazir™. The functions of these pretreatments are described as follows. The median filter was a nonlinear low-pass filter that removed high-frequency noise and preserved edges in the sample spectrum [23, 24]. Normalization normalized residuals by transforming data to reach a linear relationship between samples. Baseline correction removed baseline offsets from the spectral data [25]. SNV reduced scattering interferences or (physical) variabilities between samples (i.e. centers at a zero mean intensity and unified standard deviation) [21, 25]. In this way, SNV corrected intensities and baseline deviations due to light scattering possibly generated by impurities or density fluctuations in the samples [25]. De-trending (DT) was a polynomial baseline correction method for suppressing the baseline shifts and curvilinearity in spectra [26]. The SG derivatives removed baseline shifts and separated broad and overlapping NIR bands without significantly increasing spectral noise [21, 27].

#### Multivariate data analysis

**Fig. 1:**
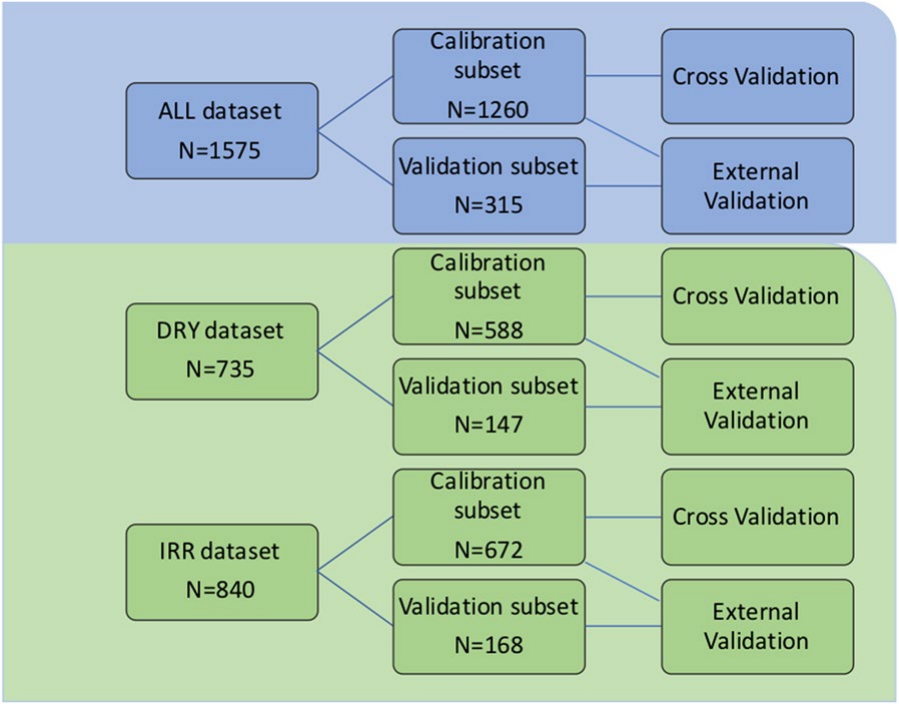
Diagram of the datasets used in cross validation (CV) and external validation (EV) processes

Validation was used for the assessment of the PLSR results. Cross-validation (CV), or internal validation, divides a dataset into several subsets (or segments) with each one containing a certain amount of samples [28]. In one epoch, the first subset of data was used for training the model, and the remaining subsets were used for model testing. For every epoch, the training and testing data subsets were different. External validation (EV), however, divides a dataset into two different complementary subsets: one for training and another one for testing [28]. In our study, CV and EV were carried out for water-stressed (DRY), non-stressed (IRR) and combined (ALL) datasets (Fig. 1). Each of the three datasets was divided into two subsets, calibration (CAL) and validation (VAL), comprising 80% and 20% of the original samples, respectively. The VAL subsets were constructed by selecting every fifth scan of each sample and were used as a test set to evaluate the robustness of the developed model. The VAL subsets were only used in EV as testing subsets while CAL subsets were used as training sets for both CV and EV. The stability and robustness of the models were improved by removing non-significant variables through the Martens’ uncertainty test during CV [29]. For the ALL dataset, 1260 and 315 data points were assigned to the CAL and VAL subsets, respectively. For the DRY dataset, CAL and VAL subsets contained 588 and 147 data points, respectively, while the IRR dataset contained 672 and 168 data points under CAL and VAL subsets, respectively. In the CV, the CAL subset was used for model training and testing, where 20, 17 and 18 segments with each segment containing 63, 34 and 37 samples were used for ALL, DRY and IRR dataset, respectively. In the EV, the CAL to VAL subsets with a ratio of 4:1 were used for model training and testing. For all the above divisions, PCA was conducted to check the effects of different irrigation conditions and the homogeneity of sub-datasets. The Unscrambler X^®^ software (v.10.5, Camo Software AS) was then used for the establishment of all the following partial least square regression (PLSR) models.

The performance of the PLSR models was determined by the following statistical parameters:

3 $${\text{R}}^{2} \left( {\text{coefficient of determination}} \right) \, = \frac{{\mathop \sum \nolimits_{i} \left( {y_{i} - f_{i} } \right)^{2} }}{{\mathop \sum \nolimits_{i} \left( {y_{i} - \bar{y}} \right)^{2} }}$$

4 $${\text{RMSE }} = \sqrt {\mathop \sum \limits_{i = 0}^{n} \left( {f_{i} - y_{i} } \right)^{2} /n}$$

where y_i_ represents the measured values and $f_{i}$ represents the predicted values. A R^2^ closer to 1 means a better fit of the measured values (y_i_) to the regression line, and root mean square error (RMSE) determines the precision of the calibration model [30].Additionally, the residual predicative deviation (RPD) was calculated as:

5 $${\text{RPD}} = \frac{\text{Standard deviation of measured extracts}}{\text{RMSE}}$$

A higher RPD value demonstrates a greater prediction power of the model [30]. In agricultural applications, especially for the materials that are more complicated in physical nature, RPD greater than 2.0 can be applied to rough screening and RPD greater than 3.0 can be interpreted as good in control quality of NIR models [31].

Finally, the optimal pretreatment approach was selected based on the above statistical parameters and used to compare the spectral data with varying wavelength ranges between two different NIR machines. An interpretation of the regression coefficients of the developed models was undertaken to determine the important chemical components contributing to rubber and resin contents. Based on this, calibration models were further upgraded using only the partial and characteristic wavelength regions from previous PLSR models.

## Results and discussion

### Rubber and resin contents

Phenotypic variations were observed for adjusted rubber and resin content in guayule accessions grown under different irrigation conditions (Table 2). In general, guayule accessions grown under stressed conditions had higher resin and rubber content compared to non-stressed conditions. The resin content of plants grown under stress conditions ranged from 8.33% to 21.03% with an average content of 13.92%, while plants grown under non-stressed conditions had resin content ranging from 5.85 to 17.44% with an average of 11.62%. Likewise, the rubber content of plants grown under stressed conditions ranged from 1.16 to 9.68% with an average of 3.94%, while under non-stressed conditions, the rubber content ranged from 0.61 to 5.84% with an average of 2.83%. The observation of higher rubber content under dry conditions coincided with previous studies [20, 32].

**Table 2:** Descriptive statistics for adjusted resin and rubber obtained from accelerated solvent extraction (ASE)

	DRY + IRR	DRY	IRR
Adjusted resin (%)			
N	1575	735	840
Mean	12.70	13.92	11.62
Max	21.03	21.03	17.44
Min	5.845	8.33	5.85
SE	0.08	0.11	0.10
SD	3.05	2.90	2.77
Adjusted rubber (%)			
Mean	3.35	3.94	2.83
Max	9.68	9.68	5.85
Min	0.61	1.16	0.61
SE	0.04	0.06	0.05
SD	1.52	1.54	1.31

N, the number of samples in the dataset; Max, maximum; Min, minimum; SD, standard deviation

### Principal component analysis

**Fig. 2:**
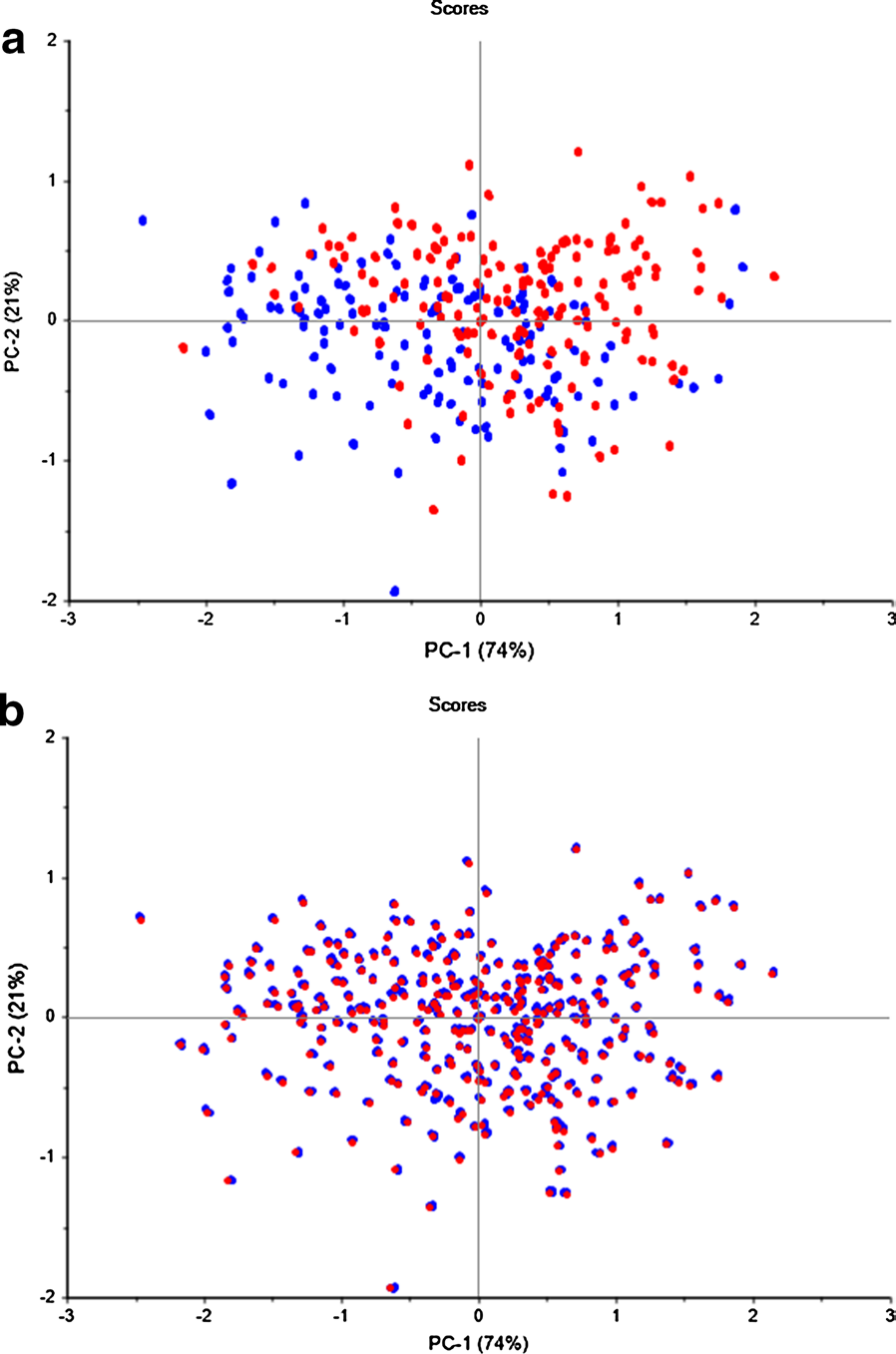
Principal component analysis (PCA) scores for two scenarios. **a** PCA distribution between DRY and IRR datasets; **b** PCA distribution between DRY and IRR datasets

The total of 1575 spectra obtained from ASD FieldSpec^®^3 were divided into two groups based on different scenarios: one was based on irrigation conditions (DRY and IRR) and another was based on calibration set (CAL) and validation set (VAL). The PCA results were shown in Fig. 2 to analyze the spectral variability between different sample groupings. The first, second and third PC accounted for 74.2%, 21.1% and 3.0% variations of raw spectral data, respectively. In total, the first three PC represented 98.3% variation of the raw spectral data. All samples in the DRY dataset distributed evenly in the IRR dataset (Fig. 2a). Likewise, all the samples in the CAL dataset distributed evenly in the VAL dataset (Fig. 2b). Thus, the division of the samples was homogenous and can be used for the following spectral analysis.

### PLSR models based on whole wavelengths

The prediction models established for resin and rubber quantification using eight different pretreatments under two different validation methods were compared in Tables 3 and 4. In general, the pretreatments improved the power and precision for rubber and resin predictions compared to no pretreatment. Even though both CV and EV concede a considerable confidence level in suppressing overfitting problems for PLRS models [33], external validation (EV) in the current study generated better calibration models than cross validation (CV) when the same pretreatment was used. This can be indicated by higher ${\text{R}}^{ 2} \left( {{\text{R}}^{ 2}_{\text{p}} > {\text{ R}}^{ 2}_{\text{cv}} } \right),$ smaller RMSE (RMSE_p_ < RMSE_cv_), smaller standard error (SEP < SECV), and higher RPD (RPD_p_ > RPD_cv_) (Tables 3 and 4). Similar results were also observed by previous studies in the estimation of biochemical methane potential (BMP) [30] and stem water potential (ψ_stem_) for the variety-specific model [33]. When under the same validation context (e.g. EV), the pretreatment combination of standard normal variate (SNV), de-trending (DT) and Savitzky–Golay 2nd derivative resulted in the most precise and robust PLSR model as compared to other pretreatments, indicating the efficiency of SNV in removing multiplicative interferences of scattering and particle size, of DT in suppressing baseline shifts and curvilinearity in diffuse reflectance spectra, and of Savitzky–Golay 2nd derivative in improving deconvolution of some overlapping spectral peaks to unveil hidden information under these peaks [30, 34]. Reliable models have also been constructed when the combinations of all or part of the three pre-processing approaches (i.e. SNV, DT, 2nd derivative) were used in previous studies [30, 35–37]. However, exceptions occurred with CV for resin content (Table 3), where the SNV + DT and smoothing + baseline + normalization resulted in the highest ${\text{R}}^{ 2}_{\text{cv}}$ values for ALL and IRR datasets, respectively. This indicated that variations may occur when the same pretreatment approach was used for different datasets or under different validation processes.

**Table 3:** Comparisons of eight different permanent approaches in the prediction of adjusted resin

Cross validation	DRY + IRR						DRY						IRR					
	${\text{R}}_{\text{CV}}^{2}$	RMSECV	RMSEC	RMSECV/RMSEC	F		${\text{R}}_{\text{CV}}^{2}$	RMSECV	RMSEC	RMSECV/RMSEC	F		${\text{R}}_{\text{CV}}^{2}$	RMSECV	RMSEC	RMSECV/RMSEC	F	
No preprocessing	0.745	1.541	1.522	1.013	7		0.777	1.367	1.333	1.026	7		0.682	1.566	1.535	1.020	7	
Smoothing	0.746	1.540	1.522	1.012	7		0.778	1.365	1.333	1.024	7		0.680	1.571	1.535	1.023	7	
Smoothing + normalization	0.740	1.556	1.537	1.012	6		0.779	1.361	1.334	1.020	6		0.686	1.556	1.521	1.023	7	
Smoothing + baseline + normalization	0.738	1.564	1.550	1.009	7		0.776	1.369	1.341	1.021	7		0.694	1.534	1.502	1.022	7	
SNV	0.746	1.539	1.526	1.009	6		0.780	1.359	1.331	1.021	6		0.686	1.555	1.522	1.021	6	
SNV + DT	0.747	1.536	1.517	1.013	7		0.788	1.334	1.295	1.030	7		0.685	1.558	1.523	1.023	6	
SNV + DT + 1st derivative	0.746	1.539	1.518	1.014	5		0.776	1.371	1.355	1.012	3		0.691	1.543	1.528	1.009	3	
SNV + DT + 2nd derivative	0.729	1.590	1.569	1.013	3		0.820	1.230	1.070	1.149	7		0.688	1.551	1.510	1.027	3	

External validation	${\text{R}}_{\text{P}}^{2}$	RMSEP	RMSEC	RMSEP/RMSEC	F	RPD	${\text{R}}_{\text{P}}^{2}$	RMSEP	RMSEC	RMSEP/RMSEC	F	RPD	${\text{R}}_{\text{P}}^{2}$	RMSEP	RMSEC	RMSEP/RMSEC	F	RPD
SNV + DT + 2nd derivative	0.764	1.486	1.437	1.034	6	2.056	0.829	1.200	1.070	1.121	7	2.413	0.765	1.343	1.297	1.036	7	2.065

Smoothing: median filter smoothing; Normalization: normalization by mean; Baseline: offset and linear baseline correction; SNV: standard normal variate; DT: de-trending, 2nd polynomial; Savitzky–Golay 1st derivative, 2nd polynomial, 23 smoothing points; Savitzky–Golay 2nd derivative, 2nd polynomial, 23 smoothing points

R^2^, coefficient of determination; RMSECV, root mean squared error of cross validation; RMSEC, root mean squares error of calibration; RMSEP, root mean squared error of prediction; CV, cross validation; EV, external validation; F, number of factors (latent values) used in calibration; RPD, ratio of performance over prediction

**Table 4:** Comparisons of eight different pretreatment approaches in the prediction of adjusted rubber

Cross validation	DRY + IRR						DRY						IRR					
	${\text{R}}_{\text{CV}}^{2}$	RMSECV	RMSEC	RMSECV/RMSEC	F		${\text{R}}_{\text{CV}}^{2}$	RMSECV	RMSEC	RMSECV/RMSEC	F		${\text{R}}_{\text{CV}}^{2}$	RMSECV	RMSEC	RMSECV/RMSEC	F	
No preprocessing	0.652	0.899	0.885	1.016	7		0.661	0.898	0.868	1.035	7		0.632	0.794	0.776	1.022	7	
Smoothing	0.653	0.897	0.885	1.013	7		0.665	0.892	0.868	1.028	7		0.629	0.796	0.777	1.026	7	
Smoothing + normalization	0.642	0.912	0.900	1.013	7		0.682	0.870	0.853	1.021	7		0.614	0.813	0.794	1.023	7	
Smoothing + baseline + normalization	0.658	0.890	0.883	1.008	7		0.658	0.900	0.868	1.037	7		0.625	0.801	0.781	1.025	7	
SNV	0.660	0.890	0.878	1.013	7		0.684	0.866	0.846	1.024	7		0.631	0.794	0.776	1.023	7	
SNV + DT	0.667	0.879	0.870	1.011	7		0.699	0.846	0.818	1.034	7		0.641	0.784	0.766	1.023	7	
SNV + DT + 1st derivative	0.683	0.857	0.846	1.014	5		0.718	0.819	0.776	1.056	7		0.667	0.755	0.740	1.020	4	
SNV + DT + 2nd derivative	0.733	0.788	0.731	1.078	7		0.756	0.763	0.661	1.153	7		0.728	0.683	0.599	1.140	7	

External validation	${\text{R}}_{\text{P}}^{2}$	RMSEP	RMSEC	RMSEP/RMSEC	F	RPD	${\text{R}}_{\text{P}}^{2}$	RMSEP	RMSEC	RMSEP/RMSEC	F	RPD	${\text{R}}_{\text{P}}^{2}$	RMSEP	RMSEC	RMSEP/RMSEC	F	RPD
SNV + DT + 2nd derivative	0.756	0.753	0.731	1.030	7	2.024	0.780	0.724	0.661	1.095	7	2.128	0.755	0.647	0.618	1.047	6	2.019

Smoothing: median filter smoothing; Normalization: normalization by mean; Baseline: offset and linear baseline correction; SNV: standard normal variate; DT: de-trending, 2nd polynomial; Savitzky–Golay 1st derivative, 2nd polynomial, 23 smoothing points; Savitzky–Golay 2nd derivative, 2nd polynomial, 23 smoothing points

R^2^, coefficient of determination; RMSECV, root mean squared error of cross validation; RMSEC, root mean squared error of calibration; CV, cross validation; EV, external validation; F, number of factors (latent values) used in calibration; RPD, ratio of performance over prediction)

#### Acetone and cyclohexane extracts (or resin and rubber)

The PLSR models for resin and rubber content were constructed by using NIR spectra obtained from rapid measurements in dry ground guayule stems. After the combination of SNV, DT and Savitzky–Golay 2nd derivative preprocessing, the R^2^ and RPD values for predicting adjusted resin were slightly higher than that for adjusted rubber (Tables 3 and 4), indicating that the models established for resin were more robust and precise than for rubber. From Tables 3 and 4, the ${\text{R}}_{\text{cv}}^{ 2}$ values for resin content were 0.729, 0.822 and 0.688 in ALL, DRY and IRR dataset, respectively, while the ${\text{R}}_{\text{p}}^{ 2}$ values were 0.764, 0.829 and 0.765 with RPD_p_ values of 2.055, 2.415 and 2.065 for the three datasets, respectively. Likewise, for rubber content, the ${\text{R}}_{\text{cv}}^{ 2}$ values after pretreatment of SNV, DT and Savitzky–Golay 2nd derivate were 0.733, 0.756, and 0.728 in ALL, DRY and IRR dataset, respectively, while the ${\text{R}}_{\text{p}}^{ 2}$ values were 0.756, 0.78 and 0.755 with RPD_p_ values of 2.024, 2.128 and 2.020 for the three datasets, respectively. The greater the RPD value is, the more reliable the model will be [31], indicating that models established for resin were more robust and reliable than for rubber, and the models established separately for the samples grown under different conditions (i.e. DRY and IRR) could better reflect and differentiate the predicting power for the traits of interests. To illustrate, under both CV and EV, the R^2^ and RPD values from the DRY dataset were higher than putting all the dry and irrigated samples together while R^2^ and RPD values from the IRR dataset were lower than the ALL dataset, meaning that putting all the samples from different growing conditions together might mitigate or weaken the predictive power and accuracy of models. Undeniably, our NIR models seem not as powerful as the ones (R^2^ > 0.95) established by previous researchers [2, 4, 6, 18, 38]; however, the previous studies on rubber-producing plants were all based on a limited number of accessions and large numbers of NIR scans, and this technical strategy might lead to overestimation of the stability and accuracy in the prediction of PLSR models. In contrast, our models were based on 56 different accessions representing a USDA guayule germplasm collection and included wild and improved genetic materials that were planted under different growth conditions [19]. Thus, these models could be more representative for general use in predicting guayule resin and rubber.

#### Comparisons between two different NIR instruments

A comparison between two commonly used NIR instruments (ASD FieldSpec^®^3 and Polychromix Phazir™) was made after the determination of the optimal pretreatment method, which was the SNV + DT + Savitzky–Golay 2nd derivate under EV context (Table 5). Not surprisingly, the ASD models with both whole wavelengths (750–2500 nm) and partial wavelengths (1100–2400 nm, 1600–2400 nm) generated significantly better predictive power than Polychromix models (1600–2400 nm), which can be seen from higher ${\text{R}}_{\text{p}}^{ 2}$ and RPD_p_ values in Table 5. This study is the first one that compares two commonly used NIR instruments for resin and rubber quantification. The better models established from ASD FieldSpec^®^3 than Polychromix Phazir™ data were probably due to different signal/noise ratio, different ways that the samples were presented during measurements, the different stability of equipments or different spectral resolutions between two instruments Bangalore et al. [44].

**Table 5:** Comparisons between models constructed using the NIR spectra from ASD FieldSpec^®^3 and Polychromix Phazir™ with various wavelengths

	DRY + IRR						DRY						IRR					
	${\text{R}}_{\text{P}}^{2}$	RMSEP	RMSEC	RMSEP/RMSEC	F	RPD_P_	${\text{R}}_{\text{P}}^{2}$	RMSEP	RMSEC	RMSEP/RMSEC	F	RPD_P_	${\text{R}}_{\text{P}}^{2}$	RMSEP	RMSEC	RMSEP/RMSEC	F	RPD_P_
Resin																		
ASD (750–2500 nm)	0.764	1.486	1.437	1.034	6	2.056	0.829	1.200	1.070	1.121	7	2.413	0.765	1.343	1.297	1.036	7	2.065
ASD (1100–2400 nm)	0.772	1.460	1.435	1.017	7	2.092	0.846	1.139	1.104	1.032	7	2.542	0.768	1.334	1.273	1.048	7	2.078
ASD (1600–2400 nm)	0.758	1.503	1.478	1.017	7	2.032	0.837	1.170	1.140	1.026	7	2.474	0.742	1.407	1.351	1.042	7	1.971
Polychromix (1600–2400 nm)	0.656	1.791	1.824	0.982	6	1.705	0.699	1.568	1.628	0.963	6	1.847	0.617	1.715	1.682	1.020	7	1.617
Rubber																		
ASD (750–2500 nm)	0.756	0.753	0.731	1.030	7	2.024	0.780	0.724	0.661	1.095	7	2.128	0.755	0.647	0.618	1.047	6	2.019
ASD (1100–2400 nm)	0.759	0.750	0.741	1.012	7	2.033	0.793	0.702	0.685	1.024	7	2.196	0.746	0.658	0.633	1.039	5	1.986
ASD (1600-2400 nm)	0.720	0.808	0.805	1.004	5	1.887	0.752	0.769	0.750	1.025	6	2.005	0.757	0.644	0.612	1.053	7	2.029
Polychromix (1600–2400 nm)	0.633	0.885	0.890	0.994	6	1.722	0.644	0.855	0.907	0.942	7	1.802	0.588	0.839	0.785	1.069	6	1.558

All the models were constructed using the optimal combination of standard normal variate (SNV) + de-trending (DT) and Savitzky–Golay 2nd derivative under external validation (EV) context

${\text{R}}_{\text{P}}^{2}$, coefficient of determination; RMSEP, root mean squared error of prediction; RMSEC, root mean squared error of calibration; F, number of factors used in calibration; RPD, ratio of performance over prediction

**Fig. 3:**
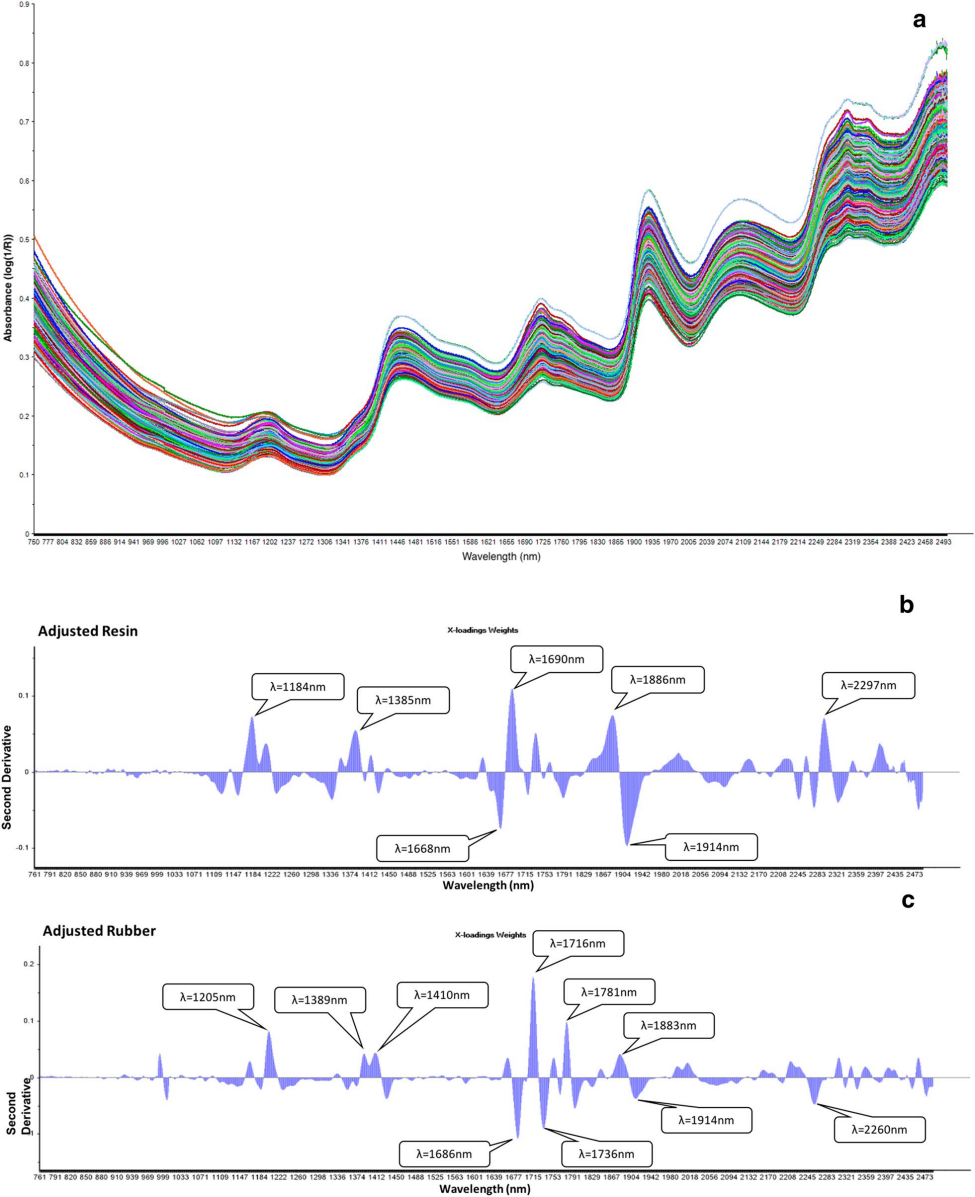

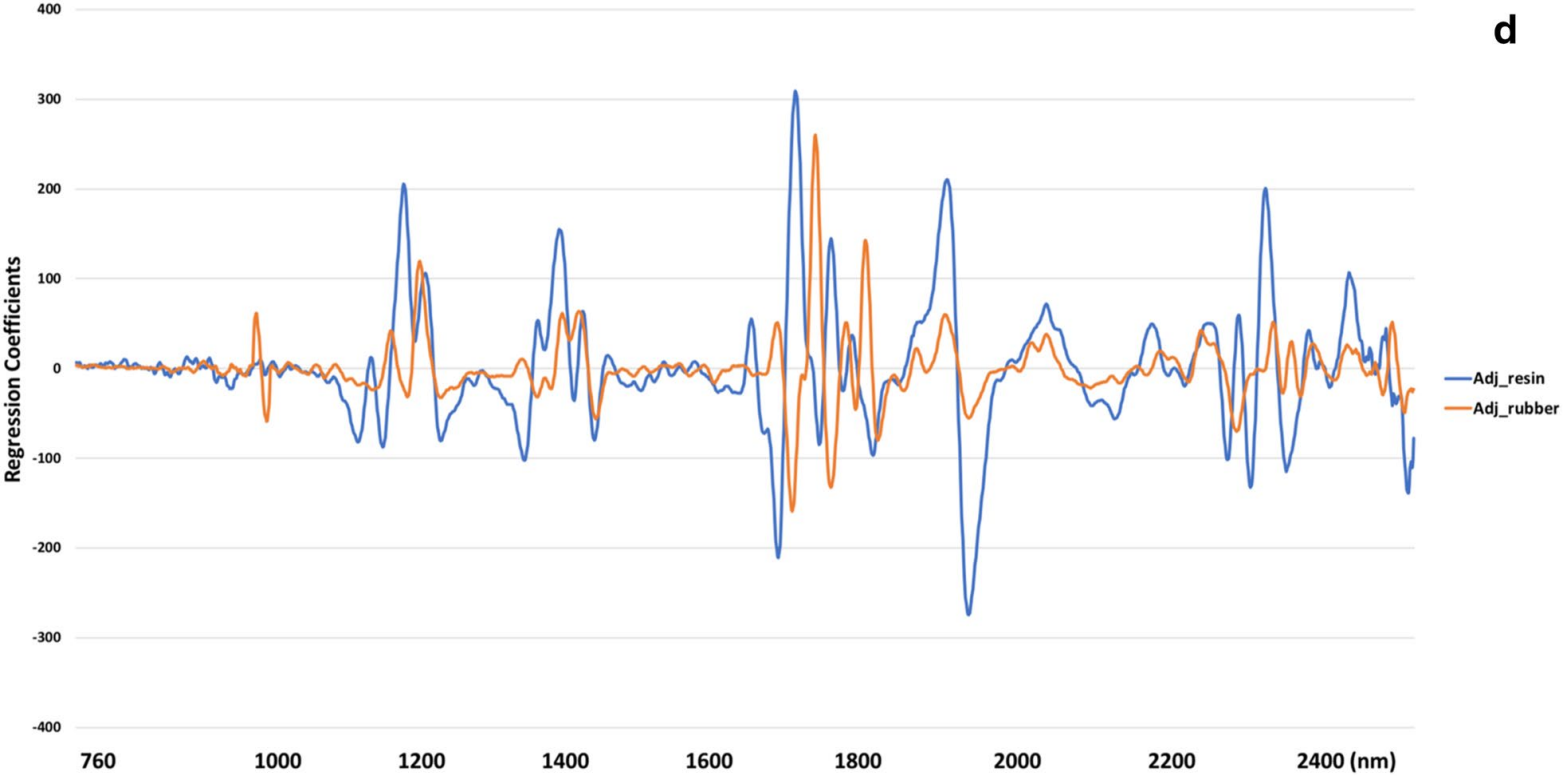
Important characteristic wavelengths contributed to partial least squares regression (PLSR) models for predicting resin and rubber content in guayule samples grown under dry condition. **a** The original absorbance spectra plot for all the scanned samples using ASD FieldSpec^®^3 spectrophotometer; **b** Second derivative of NIR reflectance spectra from ASD FieldSpec^®^3 spectrophotometer for resin content; **c** Second derivative of NIR reflectance spectra from ASD FieldSpec^®^3 spectrophotometer for rubber content; **d** Resin and rubber calibration beta coefficients from ASD FieldSpec^®^3 spectrophotometer as a function of wavelengths

In general, reflectance at different wavelengths depended on and were closely associated with the structures of chemical components. The original reflectance plot was provided (Fig. 3a). The second derivative of reflectance for guayule resin highlighted prominent peaks centered at 1184, 1385, 1668, 1690, 1886, 1914, 2248, 2278, 2297, and 2324 nm, while the sharp peaks and valleys for guayule rubber occurred at 1205, 1389, 1410, 1686, 1716, 1736, 1781, 1883, 1914, and 2260 nm (Fig. 3b, c). The similarity of prominent wavelengths for resin and rubber (Fig. 3d) was confirmed by the significant Pearson’s correlation coefficient between resin and rubber content in guayule (p = 0.038). However, this result contradicted the previous results where the absence of high correlation between resin and rubber was observed despite common loadings [2]. The NIR spectrum obtained from samples grown under different irrigation conditions (i.e., DRY and IRR), even though with varying regression coefficients across wavelengths (Additional file 1: Table S1), generated similar peaks and valleys at the above spectral regions, which showed significant contributions to the calibration models for resin and rubber content in guayule (Fig. 3b, c). The wavelength regions between 1140 and 1250 nm and 1360–1450 nm were correlated to the second overtone of C–H stretching [39], where the peaks at 1184, 1385 nm for resin and 1205, 1389, and 1410 nm for rubber were located. The wavelengths from 1640 to 1800 have been described as the first overtone of C–H stretching combination bands [40], where the peaks at 1668, 1690 nm for methyl groups in resin and 1686, 1716, 1736, 1781 nm for polyisoprenes in rubber were located. Similarly, Suchat et al. [2] and Black et al. [6] also found principle absorption bands within these ranges. Meanwhile, the bands from 2200 to 2440 nm with peaks centered at 2248, 2278, 2297 and 2324 nm for resin as well as 2260 nm for rubber could be due to the C–H stretching/C–H deformation combination [40] caused by surrounding molecules of rubber particles in guayule. In accordance, previous studies [2, 6] also identified prominent vibrations within the ranges of C–H stretch and deformation combination of CH_2_ from lipids, and C–H/C=O stretch combination of aldehyde structure [39]. This is not surprising because lipids help form one of the major membrane components surrounding rubber particles [41] and aldehydes serve as functional groups bonded to natural rubber [42]. In addition, the prominent peaks occurring at 1886 and 1914 nm for resin as well as 1410, 1883, and 1914 nm for rubber were likely to be located within the O–H stretch first overtone within the ranges of 1400–1460 nm and 1900–1960 nm, which were associated with the absorption by water molecules.

### PLSR models based on selected characteristic wavelengths

**Fig. 4:**
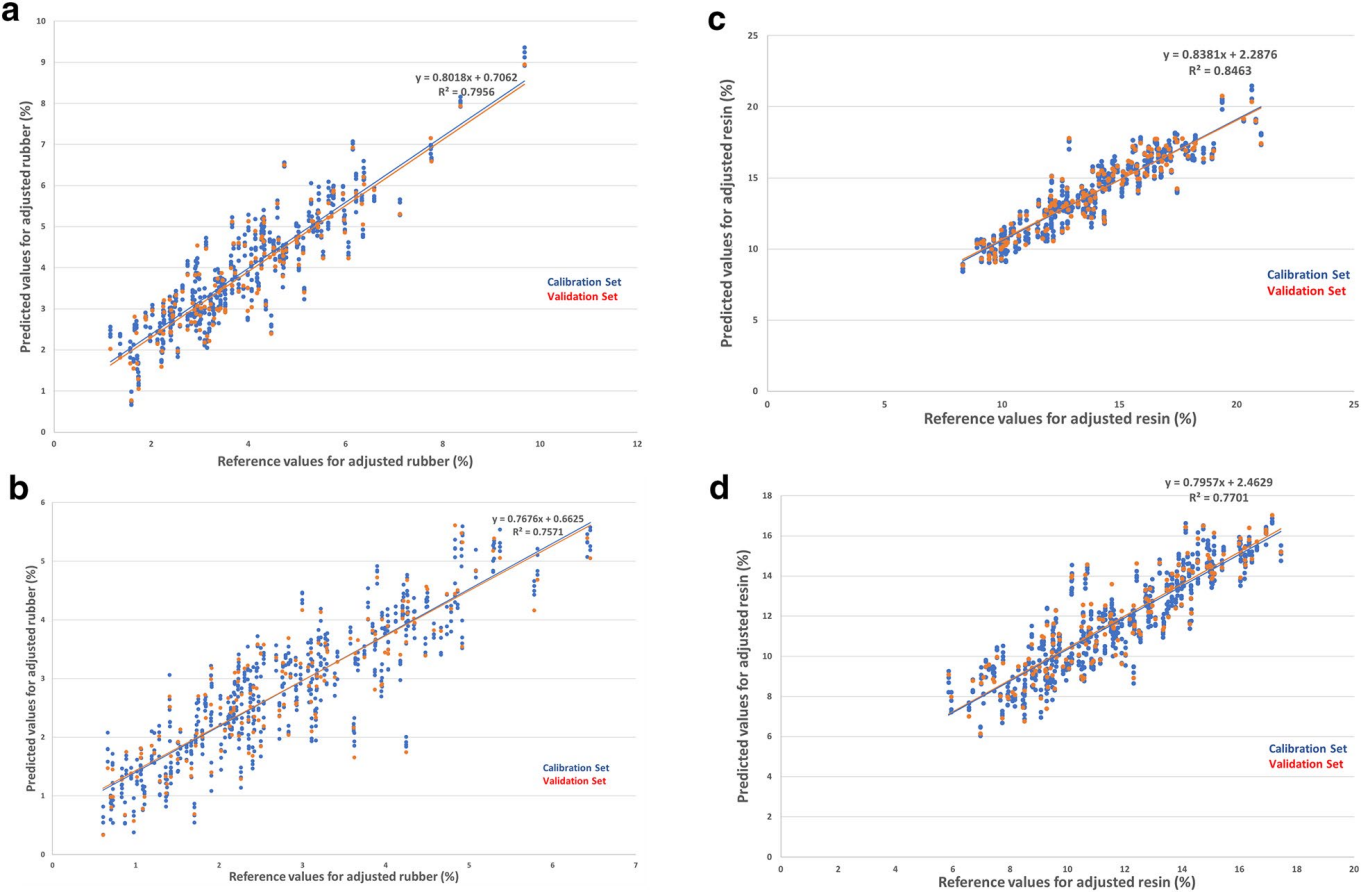
Partial least squares regression (PLSR) models predicting resin and rubber contents for guayule samples grown under different irrigation conditions based on partial wavelengths. **a** Scatter plot of ASE values versus NIR values in DRY dataset based on partial wavelengths (1100–2400 nm): rubber content. **b** Scatter plot of ASE values versus NIR values in IRR dataset based on partial wavelengths (1600–2400 nm): rubber content. **c** Scatter plot of ASE values versus NIR values in DRY dataset based on partial wavelengths (1100–2400 nm): resin content. **d** Scatter plot of ASE values versus NIR values in IRR dataset based on partial wavelengths (1100–2400 nm): resin content

The best correlative PLSR models were developed within the range of 1100–2400 nm for resin and rubber under ALL, DRY and IRR datasets except for rubber in IRR dataset, where the range of 1600–2400 nm generated the best PLSR model (Table 5, Fig. 4). With this selected range, the ${\text{R}}_{\text{p}}^{ 2}$ for resin and rubber for the DRY dataset were improved to 0.846 and 0.793 with RPD_p_ of 2.542 and 2.195, respectively. Likewise, the ${\text{R}}_{\text{p}}^{ 2}$ for resin and rubber for the IRR dataset were improved to 0.768 and 0.757 with RPD_p_ of 2.079 and 2.030, respectively. In general, the PLSR models for guayule resin and rubber for the DRY dataset were again better than the IRR dataset, and the models for guayule resin were again more powerful than rubber. However, the PLSR models based on the characteristic wavelengths (i.e. 1140–1250 nm, 1360–1450 nm, 2200–2440 nm) were slightly less powerful than those based on the partial range (1100–2400 nm) (Table 5). Selecting a few characteristic wavelengths doesn’t always help improve model prediction precision. Similar results were also observed by Kopicky [17], in which the best model was constructed within the range of 1100–1800 nm instead of the characteristic wavelengths for rubber content. However, more optimization techniques such as genetic algorithms (GA), stepwise elimination (SE), simulated annealing (SA), and generalized simulated annealing (GSA) can be implemented with PLS or internal PLS (*i*PLS) to improve the accuracy of predictions for selected characteristic wavelengths [43–45]. The principle behind *i*PLS is to split the spectra into smaller equidistant subintervals and develop PLS models on each subinterval [43, 46]. Future research is needed to further optimize the selected characteristic wavelengths.

## Conclusion

We have successfully constructed reliable high-throughput PLSR models for the determination of resin and rubber in dry, ground, guayule biomass using NIR spectroscopy. The prediction power of the models for resin content were better than rubber content and the increased spectral resolution of data from the ASD FieldSpec^®^3 improved the prediction accuracy as compared to data from the Polychromix Phazir™. Samples collected from different growing conditions are suggested to be separated for independent model establishment. In general, the established models might be used in the future to form a simple, low-cost and efficient pipeline to maximize the phenotyping efficiency in determining guayule rubber content. The established models could enable guayule breeders to efficiently screen large populations for individuals with superior traits of interests.

## Supplementary information

**Additional file 1:** Table S1. Regression coefficients across the whole NIR spectrum wavelengths obtained from samples grown under different irrigation conditions (i.e. DRY and IRR).

## Data Availability

Please contact the corresponding author for data requests.

## References

[1] Luo Z, Iaffaldano BJ, Zhuang XF, Fresnedo-Ramirez J, Cornish K (2017). Analysis of the first *Taraxacum kok-saghyz* transcriptome reveals potential rubber yield related SNPs. Sci Rep.

[2] Suchat S, Pioch D, Palu S, Tardan E, van Loo EN, Davrieux F (2013). Fast determination of the resin and rubber content in *Parthenium argentatum* biomass using near infrared spectroscopy. Ind Crop Prod.

[3] van Beilen JB, Poirier Y (2007). Guayule and Russian dandelion as alternative sources of natural rubber. Crit Rev Biotechnol.

[4] Cornish K, Myers MD, Kelley SS (2004). Latex quantification in homogenate and purified latex samples from various plant species using near infrared reflectance spectroscopy. Ind Crop Prod.

[5] Derodriguez DJ, Kuruvadi S (1991). Comparison of soxhlet and homogenizer extraction methods to determine rubber and resin content of mexican guayule plants. Bioresour Technol.

[6] Black LT, Hamerstrand GE, Kwolek WF (1985). Analysis of rubber, resin, and moisture-content of guayule by near-infrared reflectance spectroscopy. Rubber Chem Technol.

[7] Schloman WW, Carlson DW, Hilton AS (1988). Guayule extractables-influence of extraction conditions on yield and composition. Biomass.

[8] Cornish K, Pearson CH, Rath DJ (2013). Accurate quantification of guayule resin and rubber requires sample drying below a critical temperature threshold. Ind Crop Prod.

[9] Richter BE, Jones BA, Ezzell JL, Porter NL, Avdalovic N, Pohl C (1996). Accelerated solvent extraction: a technique for sample preparation. Anal Chem.

[10] Ramirez-Cadavid DA, Valles-Ramirez S, Cornish K, Michel FC (2018). Simultaneous quantification of rubber, inulin, and resins in *Taraxacum kok-saghyz* (TK) roots by sequential solvent extraction. Ind Crop Prod.

[11] Salvucci ME, Coffelt TA, Cornish K (2009). Improved methods for extraction and quantification of resin and rubber from guayule. Ind Crop Prod.

[12] Jin XL, Chen XL, Shi CH, Li M, Guan YJ, Yu CY, Yamada T, Sacks EJ, Peng JH (2017). Determination of hemicellulose, cellulose and lignin content using visible and near infrared spectroscopy in *Miscanthus sinensis*. Bioresour Technol.

[13] Lin C, Chen X, Jian L, Shi CH, Jin XL, Zhang GP (2014). Determination of grain protein content by near-infrared spectrometry and multivariate calibration in barley. Food Chem.

[14] Aernouts B, Van Beers R, Watte R, Huybrechts T, Lammertyn J, Saeys W (2015). Visible and near-infrared bulk optical properties of raw milk. J Dairy Sci.

[15] Prevolnik M, Skrlep M, Janes L, Velikonja-Bolta S, Skorjanc D, Candek-Potokar M (2011). Accuracy of near infrared spectroscopy for prediction of chemical composition, salt content and free amino acids in dry-cured ham. Meat Sci.

[16] Sparen A, Hartman M, Fransson M, Johansson J, Svensson O (2015). Matrix effects in quantitative assessment of pharmaceutical tablets using transmission raman and near-infrared (NIR) spectroscopy. Appl Spectrosc.

[17] Kopicky SE (2014). The use of near infrared spectroscopy in rubber quantification.

[18] Taurines M, Brancheriau L, Palu S, Pioch D, Tardan E, Boutahar N, Sartre P, Meunier F (2019). Determination of natural rubber and resin content of guayule fresh biomass by near infrared spectroscopy. Ind Crop Prod.

[19] Luo Z, Abdel-Haleem H (2019). Phenotypic diversity of USDA guayule germplasm collection grown under T different irrigation conditions. Ind Crop Prod.

[20] Hunsaker DJ, Elshikha DM (2017). Surface irrigation management for guayule rubber production in the US desert Southwest. Agr Water Manag.

[21] Palou A, Miro A, Blanco M, Larraz R, Gomez JF, Martinez T, Gonzalez JM, Alcala M (2017). Calibration sets selection strategy for the construction of robust PLS models for prediction of biodiesel/diesel blends physico-chemical properties using NIR spectroscopy. Spectrochim Acta A.

[22] Eide I, Westad F (2018). Automated multivariate analysis of multi-sensor data submitted online: real-time environmental monitoring. Plos ONE.

[23] Fried R, Einbeck J, Gather U (2007). Weighted Repeated median smoothing and filtering. J Am Stat Assoc.

[24] Panchuk V, Semenov V, Legin A, Kirsanov D (2018). Signal smoothing with PLS regression. Anal Chem.

[25] Delwiche SR, Reeves JB (2004). The effect of spectral pre-treatments on the partial least squares modelling of agricultural products. J Near Infrared Spec.

[26] Luypaert J, Zhang MH, Massart DL (2003). Feasibility study for the use of near infrared spectroscopy in the qualitative and quantitative analysis of green tea, *Camellia sinensis* (L.). Anal Chim Acta.

[27] Savitzky A, Golay MJE (1964). Smoothing + differentiation of data by simplified least squares procedures. Anal Chem.

[28] Rehman NU, Ali L, Al-Harrasi A, Mabood F, Al-Broumi M, Khan AL, Hussain H, Hussain J, Csuk R (2018). Quantification of AKBA in *Boswellia sacra* using NIRS coupled with PLSR as an alternative method and cross-validation by HPLC. Phytochem Anal.

[29] Martens H, Martens M (2000). Modified Jack-knife estimation of parameter uncertainty in bilinear modelling by partial least squares regression (PLSR). Food Qual Prefer.

[30] Bekiaris G, Triolo JM, Peltre C, Pedersen L, Jensen LS, Bruun S (2015). Rapid estimation of the biochemical methane potential of plant biomasses using Fourier transform mid-infrared photoacoustic spectroscopy. Bioresour Technol.

[31] Williams P (2010). The RPD statistic: a tutorial note. NIR News.

[32] Veatch-Blohm ME, Ray DT. Water stress effects on rubber concentration and rubber distribution in guayule. In: Pascual-Villalobos MJFSN, Bailey CA, Correal E, Schloman WW. Industrial crops and rural development. 2005; p. 607–7.

[33] Gutierrez S, Tardaguila J, Fernandez-Novales J, Diago MP (2016). Data mining and NIR spectroscopy in viticulture: applications for plant phenotyping under field conditions. Sensors..

[34] de Aragao BJG, Messaddeq Y (2008). Peak separation by derivative spectroscopy applied to FTIR analysis of hydrolized silica. J Brazil Chem Soc.

[35] Font R, Del Rio-Celestino M, Rosa E, Aires A, De Haro-Bailon A (2005). Glucosinolate assessment in *Brassica oleracea* leaves by near-infrared spectroscopy. J Agr Sci.

[36] Font R, Del Rio M, Velez D, Montoro R, De Haro A (2004). Use of near-infrared spectroscopy for determining the total arsenic content in prostrate amaranth. Sci Total Environ.

[37] Gonzalez-Martin I, Hernandez-Hierro JM, Bustamante-Rangel M, Barros-Ferreiro N (2006). Near-infrared spectroscopy (NIRS) reflectance technology for the determination of tocopherols in alfalfa. Anal Bioanal Chem.

[38] Takeno S, Bamba T, Nakazawa Y, Fukusaki E, Okazawa A, Kobayashi A (2008). A high-throughput and solvent-free method for measurement of natural polyisoprene content in leaves by fourier transform near infrared spectroscopy. J Biosci Bioeng.

[39] Osborne B, Fearn T, Hindle P (1993). Practical NIR spectroscopy: with applications in food and beverage analysis.

[40] Kirchler CG, Pezzei CK, Bec KB, Mayr S, Ishigaki M, Ozaki Y, Huck CW (2017). Critical evaluation of spectral information of benchtop vs. portable near-infrared spectrometers: quantum chemistry and two-dimensional correlation spectroscopy for a better understanding of PLS regression models of the rosmarinic acid content in *Rosmarini folium*. Analyst.

[41] Chan AJ, Steenkeste K, Ely M, Brssn D, Gaboriaud F, Fontaine-Aupart MP (2015). Lipid content in small and large natural rubber particles. Rubber Chem Technol.

[42] Rose K, Steinbuchel A (2005). Biodegradation of natural rubber and related compounds: recent insights into a hardly understood catabolic capability of microorganisms. Appl Environ Microbiol.

[43] Norgaard L, Saudland A, Wagner J, Nielsen JP, Munck L, Engelsen SB (2000). Interval partial least-squares regression (iPLS): a comparative chemometric study with an example from near-infrared spectroscopy. Appl Spectrosc.

[44] Bangalore AS, Shaffer RE, Small GW, Arnold MA (1996). Genetic algorithm-based method for selecting wavelengths and model size for use with partial least-squares regression: application to near-infrared spectroscopy. Anal Chem.

[45] Thorp KR, Wang G, Bronson KF, Badaruddin M, Mon J (2017). Hyperspectral data mining to identify relevant canopy spectral features for estimating durum wheat growth, nitrogen status, and grain yield. Comput Electron Agr.

[46] Chen QS, Jiang P, Zhao JW (2010). Measurement of total flavone content in snow lotus (*Saussurea involucrate*) using near infrared spectroscopy combined with interval PLS and genetic algorithm. Spectrochim Acta A.

